# Brassinosteroid Mediated Cell Wall Remodeling in Grasses under Abiotic Stress

**DOI:** 10.3389/fpls.2017.00806

**Published:** 2017-05-17

**Authors:** Xiaolan Rao, Richard A. Dixon

**Affiliations:** ^1^BioDiscovery Institute and Department of Biological Sciences, University of North Texas, DentonTX, United States; ^2^BioEnergy Science Center, US Department of Energy, Oak RidgeTN, United States

**Keywords:** cell wall, cell wall remodeling, brassinosteroid, phytohormone, abiotic stress

## Abstract

Unlike animals, plants, being sessile, cannot escape from exposure to severe abiotic stresses such as extreme temperature and water deficit. The dynamic structure of plant cell wall enables them to undergo compensatory changes, as well as maintain physical strength, with changing environments. Plant hormones known as brassinosteroids (BRs) play a key role in determining cell wall expansion during stress responses. Cell wall deposition differs between grasses (Poaceae) and dicots. Grass species include many important food, fiber, and biofuel crops. In this article, we focus on recent advances in BR-regulated cell wall biosynthesis and remodeling in response to stresses, comparing our understanding of the mechanisms in grass species with those in the more studied dicots. A more comprehensive understanding of BR-mediated changes in cell wall integrity in grass species will benefit the development of genetic tools to improve crop productivity, fiber quality and plant biomass recalcitrance.

## Introduction

During its whole life cycle, a plant’s physical survival is threatened by exposure to various biotic and abiotic stresses, which can cause morphological and physiological changes that limit growth and productivity (Bajguz and Hayat, 2009). A thin and tough layer called the cell wall surrounds plant cells to provide structural strength and act as a protective barrier against both biotic and abiotic stresses, such as pathogen attack and salinity (osmotic stress) (Taiz and Zeiger, 1998).

Phytohormones are chemical mediators that enable plants to coordinate a variety of cellular processes such as rapid responses to external stimuli (Deb et al., 2016); regulation by phytohormones is required for cell wall sensing and reconstruction during adaptive responses to adverse conditions (Didi et al., 2015; Houston et al., 2016). Brassinosteroids (BR) are a family of plant steroid hormones that elicit cell expansion (Vriet et al., 2013). Plant cell expansion and differentiation are inherently accompanied by a series of dynamic changes in cell wall composition (Hofte, 2015). The BR signaling pathway is fine-tuned to determine cell wall loosening or stiffening to assure the appropriate cell wall properties under various environmental conditions (Voxeur and Hofte, 2016). Application of exogenous BR has been proven to enhance crop tolerance to unfavorable conditions (Sharma et al., 2013), and genetic manipulation of genes that control endogenous BR levels can promote crop tolerance and improve biomass yield under a wide arrange of abiotic stress conditions (Wu et al., 2008; Ahammed et al., 2015).

The grass family (the Poaceae), one of the largest flowering plant families, covers one fifth of the earth’s land (Fincher, 2009). Grasses, including rice, maize, wheat, switchgrass, ryegrass and related species, dominate the majority of human food, livestock feed, biofuel resource and lawn and ornamental use. Grass cell walls constitute a major portion of the plant biomass and present unique features compared with those of dicots (Vogel, 2008). Therefore, understanding hormonal regulation of grass cell wall construction under adverse conditions is important for future manipulation of food and biofuel crops under climate change. Recent reviews have suggested how BR signaling underlies how plant cell walls sense pathogens and operate an active defense against pathogen attack (Underwood, 2012; Bellincampi et al., 2014; Malinovsky et al., 2014). In this review, we focus on the mechanisms of cell wall remodeling in grasses under the control of BRs as a response to abiotic stresses.

## Grass Cell Wall Structure

Plant cell walls mainly consist of the polysaccharide polymers cellulose, hemicellulose and pectin, along with lignin and a small amount of structural protein (Bashline et al., 2014). Individual cellulose microfibrils are organized to form a highly ordered (crystalline) matrix via hydrogen bonds. Hemicellulose binds to the surface of cellulose to prevent cellulose microfibrils from clasping together. Pectin and structural proteins are embedded into the cellulose-hemicellulose network to enhance the correct assembly of cell wall components and, along with lignin, to provide additional mechanical strength (Taiz and Zeiger, 1998; Cosgrove and Jarvis, 2012).

There are two major types of cell wall according to composition and structure. Type I walls consist of a xyloglucan matrix into which cellulose microfibrils are embedded, with high levels of pectin and structural proteins and low levels of arabinoxylans, glucomannans, and galacto-glucomannans. In contrast, type II walls have a cross-linked network of glucuronoarabinoxylans bound to cellulose fibers, with various minor portions of pectin and structural proteins (Vogel, 2008). Type I cell walls are present in dicots, non-grass monocots and gymnosperms, and type II cell walls are present in grasses (Fincher, 2009). Additional notable features of grass cell walls include the presence of mixed-linkage β-glucans and the abundance of xylan and lignin deposited in secondary cell walls (Vogel, 2008). These unique aspects indicate the existence of genes specifically involved in grass cell wall biogenesis, many of which remain to be explored (Lin et al., 2016).

## Damage to Cell Walls Under Abiotic Stress

Among abiotic stresses, water-deficiency through drought, osmotic stress and salinity are the most challenging for crop growth and food production (Tenhaken, 2015; Feng et al., 2016). The water potential of the plant cell is in accordance with that of the environment surrounding the cell (Bray, 2007). Drought, osmotic stress and salinity decrease the water potential of the soil solution and water leaks from the cell to the external solution (Bray, 2007). As a consequence, this can lead to reduction of cell turgor and physical damage to the cell wall including disconnection of binding sites for wall components, loss of fragments from the wall, and decreased associations between the wall and the plasma membrane (Hamann, 2015b).

Another common reaction in plant responses to many stress conditions (such as heavy metal ions) is the generation of a burst of reactive oxygen species (ROS), which results in the toxicity of oxidative stress (Mittler, 2002). A rapid accumulation of ROS in plant cells inhibits the activity of antioxidants and antioxidative enzymes and can cause the degradation of lipids and even destruction of the cell membrane (Das and Roychoudhury, 2014). Moreover, the generation of OH by the Fenton reaction involving heavy metal ions or antioxidative enzymes is considered to cause plant cell wall loosening via breaking cross-linkages between ferulates and lignin (Karkonen and Kuchitsu, 2015).

A group of plasma membrane-localized receptor-like kinases (RLKs) and mechanosensitive ion channels (such as Ca^2+^ channels) are considered to directly or indirectly detect the impairment of cell wall integrity as a mechanical cue to sense adverse effects in the environment. They subsequently translate the physical changes in the cell surface to cellular signals (such as Ca^2+^ influx), which further trigger corresponding cascades of plant defense responses for stress management (Hamann and Denness, 2011; Feng et al., 2016). These responses are better understood in yeast than in plants (Hamann and Denness, 2011).

## Transduction of BR Signaling in Grasses

**FIGURE 1:**
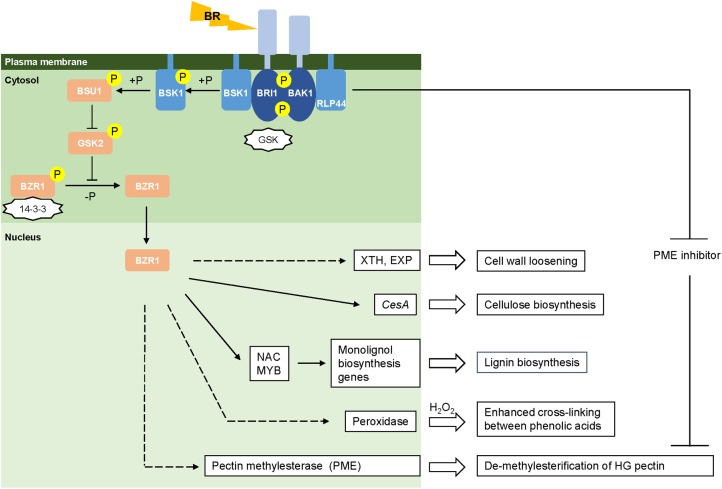
**Brassinosteroid mediated cell wall remodeling.** Models of the BR signaling pathway are drawn based on the information from rice (Vriet et al., 2013). Activated and repressed reactions are represented by arrows and blunt-ended lines, respectively. Negative regulators (GSK and 14-3-3) are in the shape of stars. Phosphorylation and de-phosphorylation are represented by “+P” and “-P,” respectively. Direct and indirect regulation are represented by solid and dashed lines, respectively.

When perceiving environmental cues, plants translate them into physiological signals through coordination of the levels of phytohormones such as gibberellic acid (GA), abscisic acid (ABA), and BRs that will further trigger cellular responses to maintain integrity and remodeling of the cell wall (Ahammed et al., 2015). Recent findings have characterized the molecular machinery of BR signal reception and transduction in Arabidopsis (Wolf et al., 2012, 2014; Zhu et al., 2013). However, the BR signaling pathway in monocots still remains to be explored, although a few conserved components have been identified in rice (Yamamuro et al., 2000; Bai et al., 2007; Li et al., 2009; Tong et al., 2012; Vriet et al., 2013; Zhang et al., 2014; Zhang B. et al., 2016), maize (Zhang Y. et al., 2016) and *Brachypodium distachyon* (Feng et al., 2015). Here, we show a proposed model for the BR signaling pathway in grasses generally based on the information for rice (**Figure 1**). BR signaling is perceived by cells through its binding to the extracellular domain of a plasma membrane-bound receptor kinase, BRASSINOSTEROID INSENSITIVE 1 (OsBRI1) (Yamamuro et al., 2000; Zhu et al., 2013). BR-binding prevents BRI1 from associating with the negative regulator OsGSK and promotes BRI1 to interact with the co-receptor kinase BRI1-ASSOCIATED RECEPTOR KINASE1 (OsBAK1) (Li et al., 2009; Zhang et al., 2014). The *trans*-phosphorylation between OsBRI1 and OsBAK1 activates the kinase activity of BRI1, the intracellular domain of which initiates the signal transduction cascade within cells (Vriet et al., 2013). BR SIGNALING KINASE (BSK1) located in the cytoplasm is phosphorylated and activated by BRI1 leading to the activation of BRI1 SUPPRESSOR1 (BSU1) (Zhang B. et al., 2016). Through desphosphorylation, BSU1 negatively regulates OsGSK2, an inhibitor of the BRASSINAZOLE-RESISTANT1 (BZR) family of transcription factors (Tong et al., 2012). Upon BR signaling, OsBZR1 is activated by dephosphorylation and inhibition of its interaction with 14-3-3 proteins. This leads to a rapid accumulation of OsBZR1 in the nucleus to directly control the expression of BR target genes (Bai et al., 2007). In Arabidopsis, the BR-activated transcription factor BZR1 and its homologous gene BZR2/BES1 have been shown to directly bind to promoter regions of a large number of cell wall-related genes (Jiang et al., 2015), including the majority of cellulose synthase genes (Xie et al., 2011), and NAC and MYB transcription factors associated with regulatory pathways for lignin synthesis (Zhao and Dixon, 2011; Benatti et al., 2012). Though direct evidence is absent, grasses may operate a similar BR-mediated signal cascade to regulate the expression of genes involved in cell wall biogenesis.

## BR-Mediated Cell Wall Remodeling

Recent evidence suggests the association of BR signaling pathways with cell wall remodeling. Here, we discuss effects of BRs on cell wall loosening proteins and major structural cell wall components including cellulose, lignin and pectin, according to recent advances in both grasses and dicots.

### BR-Mediated Cell Wall Loosening Proteins

Modification of the structure of the plant cell wall is required as a defense response upon the perception of abiotic stresses (Tenhaken, 2015). Two groups of enzymes, xyloglucan transferase/hydrolase (XTHs) and expansins, are involved in cell wall loosening. XTHs catalyze the internal cleavage of xylogulcan polymers and transfer the newly generated ends to other xyloglucan chains (Uozu et al., 2000; Eklof and Brumer, 2010), whereas expansins loosen the linkages between cellulose microfibrils through non-covalent rearrangement of their targets (Yennawar et al., 2006). A subset of XTH and expansin genes is significantly up-regulated by BL treatment in Arabidopsis and soybean (Zurek and Clouse, 1994; Kozuka et al., 2010; Abuqamar et al., 2013). Similarly, the expression of a number of XTHs and expansin genes has been reported to be regulated by BR in rice, maize and wheat (Uozu et al., 2000; Yokoyama et al., 2004; Liu et al., 2007; Genovesi et al., 2008). Considering that grasses contain lower levels of xyloglucan in their cell walls than do dicots, it has been suggested that XTH isoforms in grass species may contribute to building xyloglucan-(β-1,3:1,4-glucan) links, rather than rearrangement of xyloglucan chains (Eklof and Brumer, 2010). BR-mediated regulation of XTH and expansin mRNA levels may lead to alteration of the interaction between xyloglucan and cellulose microfibrils to alter cell wall stiffness.

### BR-Mediated Cellulose Deposition

Cellulose microfibrils, composed of β-1,4-glucan chains (Hill et al., 2014), contribute to the majority of plant above-ground biomass and their synthesis and deposition is responsive to changing environmental conditions (Wang et al., 2016). Cellulose synthesis requires multiple members of the cellulose synthase (*CesA*) gene superfamily, which encode catalytic subunits that form hexameric complexes localized on the plasma membrane (Hill et al., 2014). In Arabidopsis, BR signaling has been shown to increase cellulose accumulation through upregulation of *CesA* genes at both the transcriptional and post-transcriptional levels. The expression of most *CesA* genes is induced by BR-mediated activation of the transcription factor BES1, which directly binds to the CANNTG E-box in the promoter region of *CesA* genes (Xie et al., 2011), while the activity of CESA1 kinase is increased by the degradation of its inhibitor protein BRASSINOSTEROID INSENSITIVE2 (BIN2) (Sánchez-Rodríguez et al., 2017). Some observations suggest that grasses may share a similar BR-mediated pathway for *CesA* gene regulation. The BR receptor kinase gene *OsBRI1* shows co-expression with *OsCESA3* in a genome-scale gene network for rice (Lee et al., 2011). An associated up-regulation of *BRI1*, *CESA3* and other genes involved in BR signaling is observed in a wild wheat species (*Agropyron elongatum*) compared with domesticated genotypes during water stress. The enhanced BR-signaling pathway in *A. elongatum* may contribute to its higher water-stress tolerance and significant increase of root and shoot biomass compared with the domesticated line under water-deficient conditions (Placido et al., 2013).

Either exogenous application of BRs or overexpression of BR receptor genes could benefit cellulose deposition and accumulation, especially to compensate for cellulose loss caused by abiotic stresses (Sun et al., 2005; Li et al., 2009; Zhang et al., 2014). Some evidence has suggested that BR signaling may not directly determine the total content of cellulose (Schrick et al., 2012) but rather be more involved in the orientation of cellulose microfibril deposition through the control of the cortical microtubular organization in cells (Bashline et al., 2014).

### BR-Mediated Lignin Accumulation

The second most abundant carbon sink in plants, lignin is absent from the primary cell wall and deposited in the secondary cell wall surrounding specific cell types to enhance cell wall rigidity and provide structural support (Boerjan et al., 2003; Karkonen and Koutaniemi, 2010). Lignin is a phenolic heteropolymer, which mainly consists of three types of 4-hydroxycinnamyl alcohol units, guaiacyl (G), syringyl (S) and *p*-hydroxyphenyl (H), derived from the monolignols coniferyl alcohol, sinapyl alcohol and *p*-coumaryl alcohol, respectively (Boerjan et al., 2003; Chen et al., 2012). An induction of lignin biosynthesis is often observed under biotic and abiotic stresses as a defense response (Dixon and Paiva, 1995; Moura et al., 2010). For example, excess heavy metal (Cu, Zn, Al) causes an elevated accumulation of lignin in cell walls of rice and wheat (Moura et al., 2010).

Brassinosteroids have been reported to play a crucial role in secondary cell wall deposition. Application of the BR biosynthesis inhibitor (BRz) in cotton ovules causes severe inhibition of secondary cell wall development in the fibers (Sun et al., 2005). Tracheary element formation and secondary cell wall thickening can be observed in suspension cell cultures of Arabidopsis and banana following exogenous BR-supplementation (Oda et al., 2005; Negi et al., 2015). Furthermore, loss of function of a BR biosynthesis protein (DIM1) in Arabidopsis leads to a significant reduction in lignin content and a lower lignin S/G ratio (Hossain et al., 2012). Consistent with this finding, BR treatment induces the accumulation of lignin with predominantly S units in switchgrass suspension cells (Shen et al., 2013). A regulatory mechanism for BR signaling and secondary cell wall development has been proposed in Arabidopsis; the BR-activated transcription factor BES1 promotes the expression of VND6 and VND7, which determine the transition of xylem cells to form tracheary elements, and alters the expression of MYB transcription factors involved in regulating lignin biosynthesis (Zhong et al., 2008; Yamaguchi et al., 2010; Zhao and Dixon, 2011; Didi et al., 2015; Li et al., 2016).

Besides the regulation of genes involved in monolignol biosynthesis, BRs may also have effects on the bonds between monolignol polymers and phenolic acids in the cell wall through controlling antioxidant enzymes at the transcriptional and post-transcriptional level. The exogenous application of BR significantly increases the activity of antioxidant enzymes (such as catalase, superoxide dismutase, ascorbate peroxidase, and peroxidase) through up-regulation of the expression of the corresponding genes in maize, wheat, and rice exposed to metal stress (Vardhini and Anjum, 2015; Yan et al., 2015; Sharma et al., 2016). Peroxidases mediate the formation of phenolic radicals, leading to both lignin polymerization and cross-linking between the ferulic acid units esterified to arabinoxylans which occur especially in grasses (Hamann, 2015a; Tenhaken, 2015). The increased activity of peroxidases and the formation of ROS together enhance the covalent cross-linking of components in the cell wall and strengthen the mechanical properties of the wall (Lamb and Dixon, 1997; Tenhaken, 2015). Therefore, it is possible that BRs enhance the antioxidant defense system as well as increasing the cross-linking of phenolic compounds in the cell wall to alleviate oxidative damage caused by the ROS burst (O’Brien et al., 2012).

### BR-Mediated Pectin Modification

Pectins play a critical role in enabling cell walls to remain firm but extensible (Harholt et al., 2010). Pectic polysaccharides bind to the cellulose and hemicellulose network, forming hydrated gels to inhibit collapse of the cellulose matrix and to monitor changes in polymer residues and pH (Harholt et al., 2010; Voxeur and Hofte, 2016). Pectic polysaccharides consist of various galacturonic acid (GalUA)-containing polymers, including homogalacturonan (HG), xylogalacturonan (XGA), rhamnogalacturonan I (RGI), and rhamnogalacturonan II (RGII) as backbone units, of which GalUA residues can be substituted by arabinan, galactan, and arabinogalactan as branch chains (Harholt et al., 2010; Hofte, 2015). The degree of methylesterification in HGs determines looseness1 the stiffness of the pectic matrix and is precisely controlled by the balance of activity between pectin methylesterase enzymes (PMEs) and PME inhibitors (PMEIs) (Wolf et al., 2012). PMEs de-methyl esterify HG chains in the cell wall, which leads to a decrease in stiffness of the wall and acceleration of cell growth under Ca^2+^ limited conditions (Hofte, 2015) or promotes the formation of a HG-Ca^2+^ gel to lock the cell wall into an inextensible state under Ca^2+^ abundant conditions (Cosgrove, 2016). Ca^2+^ fluxes/levels complement BR signaling by contributing to the fine-tuned control of cell wall integrity under normal or adverse conditions. For example, BRs have been shown to upregulate the level of one PME transcript and trigger PME activity to increase the stiffness of cell walls in response to cold and freezing in Arabidopsis (Qu et al., 2011). The BR-receptor kinase BAK1 in Arabidopsis can directly interact with a plasma membrane receptor-like protein (RLP44) to repress the activity of PME inhibitors and therefore reduce the stiffness of the pectic matrix and promote cell wall loosening under both normal and stress conditions (Wolf et al., 2012, 2014). Therefore, BR signaling in Arabidopsis is coupled with the modification of methyl-esterified HGs to control pectin-dependent cell wall integrity (Wolf et al., 2012). Knowledge of BR-meditated pectin methylesterase activity so far is lacking in grasses and is an important area for future research.

## Conclusion

The possible roles of BR signaling that contribute to cell wall remodeling are summarized in **Figure 1**. Few BR response targets have been established and much remains to be discovered about how BRs regulate the expression of cell wall related genes and corresponding enzymatic activity in grasses. In addition, the crosstalk between BRs and other phytohomones in controlling cell wall integrity is another area that requires more investigation (Bai et al., 2012; Huang et al., 2015; Deb et al., 2016). A better understanding of BR-mediated cell wall homeostasis will guide the design of genetic modification strategies to improve biomass and stress tolerance in grasses.

## Author Contributions

XR collected data from literature and wrote the manuscript. RD revised the article.

## Conflict of Interest Statement

The authors declare that the research was conducted in the absence of any commercial or financial relationships that could be construed as a potential conflict of interest.
